# Sialylated Immunoglobulins for the Treatment of Immuno-Inflammatory Diseases

**DOI:** 10.3390/ijms21155472

**Published:** 2020-07-31

**Authors:** Yuliya V. Markina, Elena V. Gerasimova, Alexander M. Markin, Victor Y. Glanz, Wei-Kai Wu, Igor A. Sobenin, Alexander N. Orekhov

**Affiliations:** 1 Laboratory of Cellular and Molecular Pathology of the Cardiovascular System, Institute of Human Morphology, 3 Tsyurupy Street, 117418 Moscow, Russia; alexander.markin.34@gmail.com (A.M.M.); viglanz@outlook.com (V.Y.G.); igor.sobenin@gmail.com (I.A.S.); a.h.opexob@gmail.com (A.N.O.); 2 Department of Systemic Rheumatic Diseases, V.A. Nasonova Research Institute of Rheumatology, 34A Kashirskoe Shosse, 115522 Moscow, Russia; gerasimovaev@list.ru; 3 Department of Internal Medicine, National Taiwan University Hospital, Bei-Hu Branch, Taipei 108, Taiwan; weikaiwu0115@gmail.com; 4 Laboratory of Medical Genetics, Institute of Experimental Cardiology, National Medical Research Center of Cardiology, 15A 3-rd Cherepkovskaya Street, 121552 Moscow, Russia; 5 Laboratory of Angiopathology, Institute of General Pathology and Pathophysiology, 8 Baltiyskaya Street, 125315 Moscow, Russia; 6 Institute for Atherosclerosis Research, Skolkovo Innovative Center, 121609 Moscow, Russia

**Keywords:** sialylation, sialidase, immunoglobulins, inflammation, immuno-inflammatory diseases, atherosclerosis

## Abstract

Immunoglobulins are the potent effector proteins of the humoral immune response. In the course of evolution, immunoglobulins have formed extremely diverse types of molecular structures with antigen-recognizing, antigen-binding, and effector functions embedded in a single molecule. Polysaccharide moiety of immunoglobulins plays the essential role in immunoglobulin functioning. There is growing evidence that the carbohydrate composition of immunoglobulin-linked glycans, and especially their terminal sialic acid residues, provide a key effect on the effector functions of immunoglobulins. Possibly, sialylation of Fc glycan is a common mechanism of IgG anti-inflammatory action in vivo. Thus, the post-translational modification (glycosylation) of immunoglobulins opens up significant possibilities in the diagnosis of both immunological and inflammatory disorders and in their therapies. This review is focused on the analysis of glycosylation of immunoglobulins, which can be a promising addition to improve existing strategies for the diagnosis and treatment of various immuno-inflammatory diseases.

## 1. Introduction

Immunoglobulins, the potent effector proteins of the humoral immune response, possess both anti-inflammatory and proinflammatory activities triggered by antigen recognition based on an affinity for various fragment crystallizable receptors (FcRs) and complement factors [1,2]. Starting with unicellular and multicellular organisms, antimicrobial peptides have been crucial to the survival of cells and organisms, through cellular communication and the fight against pathogens. They are essential for the clearance of microbes by bridging the immune systems but can also partake in autoimmune disease development when generated against themselves [3]. One possible scenario of IgG-mediated inflammatory autoimmunity is reduced elimination of cell remains, which leads to diminished self-tolerance and induces an autoimmune response. B-cells act as proinflammatory agents via generation of IgG autoantibodies (aAbs). In the course of evolution, immunoglobulins have formed extremely diverse types of molecular structures with antigen-recognizing, antigen-binding and effector functions embedded in a single molecule [4]. Immunoglobulin antibodies consist of two identical sets of heavy and light chains that are interconnected by disulfide bonds and form an antigen-binding (Fab) part and an effector fragment crystallizable (Fc) part; the isotypes of IgM, IgG, IgA, IgD, and IgE are determined by five classes of conservative heavy chain domains. IgG can be divided into IgG1, IgG2, IgG3, and IgG4 subclasses, with every subclass possessing its own biological properties; IgA can similarly be divided into subclasses IgA1 and IgA2 [5]. The arrangement of disulfide bonds between the different chain types determines a well-known Y-shape, containing a hinge-type stabilizing region, which varies among Fc variants [6]. It is necessary to note that immunoglobulins belong to glycoproteins, for example, they are glycosylated, and thus bear polysaccharide moiety which plays an essential role in immunoglobulin functioning. Currently, the most studied are the glycans of IgG. The IgG Fc region contains one highly conserved N-linked glycosylation site at asparagine 297 (Asn297) in every heavy chain CH2 domain and this site is exclusively occupied by complex-type biantennary N-glycans [7]. IgG N-linked glycans share a common pentasaccharide “nucleus” linked with galactose residues, N-acetylglucosamine, terminal sialic acids, and modified by fucose. These variant terminal endings provide an extremely high heterogeneity of N-linked glycan; more than 30 glycans with different sequences of saccharide residues have been identified in circulating IgG in humans [8]. It is becoming apparent that the carbohydrate composition of Fc-linked glycans, and especially their terminal sialic acid residues, provides a key effect on the effector functions of IgG [9,10,11]. It is worthwhile noting that sialylated IgGs having an anti-inflammatory activity is a phenomenon that has been reported by numerous researchers [12,13,14]. Thus, sialylation of Fc glycan is a common anti-inflammatory mechanism of the action of IgG in vivo [8,15,16,17]. This review is focused on the analysis of glycosylation of immunoglobulins, which can be a promising addition to improve existing strategies for the diagnosis and treatment of various immuno-inflammatory diseases.

## 2. Glycosylation of Immunoglobulins: The Effects in Various Diseases

Functionally, an antibody consists of the following two parallel-evolving separate protein domains: A fragment responsible for binding an antibody (Fab, fragment antigen binding) and a fragment interacting with the cell surface Fc receptor and certain complement system proteins, i.e., a crystallized antibody domain (Fc, fragment crystallizable region). The unique ability of antibodies to recognize antigens is provided by the mechanism of structural rearrangement of the antigen binding domain through somatic recombination and hypermutation of coding regions [18]. Due to the Fc domain binding ability, the implementation of certain physiological effects of immunoglobulins is ensured (cell lysis; mast cells, basophils, and eosinophils degranulation; and opsonization) [19].

Light and heavy chains have a variable (V) N-terminal region (VL or VH) containing three hypervariable regions called the complementarity-determining regions (CDRs), as well as four framework regions. Three heavy chain CDRs and three light chain CDRs form an antigen binding domain [20,21]. The heavy chain constant domains are rearranged to modulate effector activities (complement activation or binding to Fc receptors) with unchanged antigen specificity [6,22]. However, in addition to the genomic selection of heavy chains, controlling the variation of the Fc domain, there is a second mechanism, namely glycosylation. This modification determines the functional potential of the antibody by determining the antibody Fc region and changes the affinity of antibodies to Fc receptors. As supported by numerous experimental data, glycosylation is the central actuating mechanism of the immune system [23]. Genes and the environment both influence the composition of IgG glycans which makes it an excellent biomarker of the general state of human health, and, actually, of biological age. Glycosylation of antibodies is under strict control, but in the case of dysregulation, for example, with aging or in various diseases, the changes in glycosylation patterns are observed.

Glycosylation, the process of protein modification during and after translation, affects proteins’ properties and functions. Protein glycosylation is a very dynamic post-translational modification affecting cell surface-level molecular interactions, and secreted proteins activity. The aspects influenced by glycosylation are essential and involve secretion, solubility, stability, binding, conformation changes, and antigenic properties [24]. Immunoglobulins are key serum proteins to show changes in their glycosylation pattern; these changes can, in turn, affect their immunoeffector functions [25]. Glycans make up to 15% of the weight of IgG and are the integral part of the molecule [26].

Joining of glycans with proteins occurs via one of two bonds, either at asparagine residues (N-glycans) or at serine/threonine residues (O-glycans). In contrast to other proteins that are able to interact with glycans, IgG glycans are represented by a rather conservative composition of N-linked sugar molecules [27].

**Figure 1:**
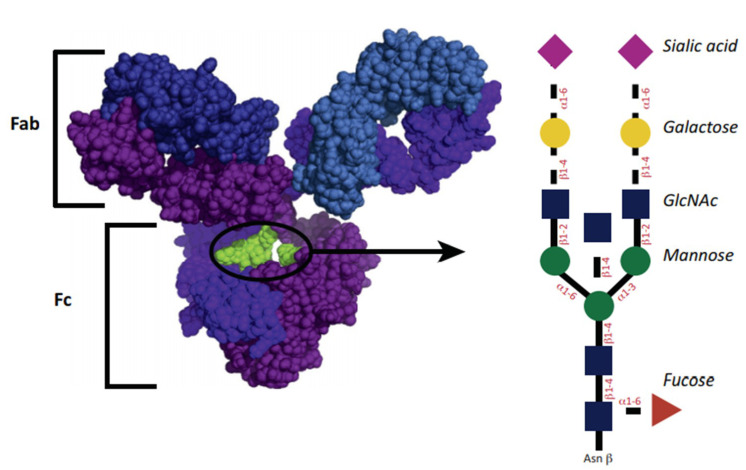
Antibody and glycan structure. Reprinted from [27], reprinted with the permission of Elsevier.

The structure of IgG glycosylation is shown in Figure 1. The glycosylation site for IgG1 is asparagine-297 belonging to the Fc domain. The glycan of the antibody is represented by a heptasaccharide consisting of two n-acetylglucosamine residues (GlcNAc) (blue squares), mannose (green circle), branching of 1,3 and 1,6 mannose, and one extra GlcNAc residue for each mannose. Additional fucoses (red triangle) are variously presented. Residues of GlcNAc can additionally join mannose, and then up to two galactoses for each residue (yellow circle), and up to two terminal sialic acids (purple triangle). A similar structure leads to some heterogeneity of antibodies [28]. The diversity of the resulting glycans affects their functional abilities [29]. Glycans can be classified by the number of galactose residues, i.e., no galactose residues (G0), one galactose residue (G1), or two galactose residues (G2). Interestingly, in inflammatory diseases, the number of non-galactosylated (agalactosylated, G0) glycans increases [29]. Conversely, an increased level of galactosylation is associated with a decrease in the inflammatory activity of antibody preparations [30,31].

In general, changes in galactosylation have been observed in many physiological conditions of a human. In particular, the accumulation of agalactosylated antibodies has been observed in inflammatory diseases (autoimmune disorders, infections, and malignant neoplasms), whereas increased galactosylation has been associated, for example, with pregnancy [32].

In addition, specific glycan changes are used in the field of monoclonal antibody therapy, thus, indicating the important role of glycans in the formation of the antibody effector function. For example, removal of fucose significantly increases the activity of the antibody-dependent cellular cytotoxicity system, whereas removal of GlcNAc alone has a more modest effect. Furthermore, sialylation reproducibly enhances the in vivo anti-inflammatory effect of immunoglobulin administered intravenously [28].

The actual glycosylation of general or antigen-specific IgG is changed in many autoimmune diseases [33]. Studying IgG composition of glycans with denaturation-based methods revealed a correlation of hypoglycosylation of IgG aAbs and proinflammatory immune responses. Alteration of glycosylation in IgG has been demonstrated for several serum-derived proteins in inflammatory arthropathies patients [34]. Studies of rheumatic disease-associated IgG glycosylation changes have revealed reduced levels of galactosylated IgG glycoforms and decreased galactosylation and sialylation in rheumatoid arthritis (RA) patients in contrast to healthy controls [34]. Among patients with RA, the presence of IgG agalactosyl glycoforms indicated a disease progression [35]. Moreover, agalactosyl IgG aAbs are pathogenic, as demonstrated under conditions of experimental collagen-induced arthritis (CIA), where the passive transfer was more effective at inducing arthritis by using the agalactosyl glycoforms of IgG aAbs to type II collagen [36]. Thus, similar to the abovementioned CIA, the evidence from patients with RA indicates that galactosylation of disease-specific aAbs has a role in the pathophysiology of RA.

Many studies have suggested that the development of certain autoimmune diseases was associated with a decrease in the overall level of IgG sialylation. In the cases of individual nosologies, a relationship has been found between total IgG or IgG autoantibodies decreased sialylation and the pathological activity of autoantibodies [37], and with severity of disease symptoms [38], its activity and course [39,40], and the response to treatment [41,42].

Sialylation is associated with an anti-inflammatory effect. An intravenous immunoglobulin used to treat inflammatory conditions has an anti-inflammatory effect due to a population of antibodies carrying 2,6-sialylated Ig glycans [8,43].

Contrary to autoimmune and alloimmune diseases, in which antibodies are usually involved in pathogenesis, they are called upon to exert protective activities. In most studies devoted to the study of IgG glycosylation levels in plasma/serum in infectious diseases, a decrease in galactosylation has been found, for example, there were distinct antigen-specific IgGs in hepatitis B and C that showed a specific glycosylation profile, which included a decrease in galactosylation, the degree of which was associated with the severity of the disease and liver damage [44]. The anti-envelope antibodies to human immunodeficiency virus (HIV) showed a decrease in galactosylation, sialylation, and fucosylation levels, as well as a decrease in total IgG galactosylation [45]. After vaccination against tetanus, influenza, pneumococcal, and meningococcal infections, antigen-specific IgG had an increased level of sialylation, galactosylation, and fucosylation of the nucleus as compared with the total IgG [46,47,48].

Variability of the Ig glycosylation profile was found in patients with cancer. Modifications of IgG glycans were first detected in patients with multiple myeloma, a malignant tumor of plasma cells related to paraproteinemic leukemia. In this case, decreased IgG galactosylation was observed as compared with healthy controls [49,50]. In addition, increased levels of terminally sialylated immunoglobulin glycans were found in multiple myeloma [51]. Moreover, the IgG glycosylation profile was specific for one line of antibody-producing B-cell clones, and for different myeloma cells, the glycosylation profiles differed [52]. One recent study showed that the differences in IgG glycosylation were disease stage dependent, and with the response to therapy [53]. Cancer studies of IgG glycosylation have shown that the amount of agalactosylated IgG was associated with disease progression and metastasis [54,55,56,57,58].

Importantly, some changes in the structure of glycans IgG can be associated with a higher risk of developing cardiovascular diseases, while other modifications can reduce CVD risk. The first group includes glycans with three GlcNAcs (GP6) or glycans with GlcNAc and sialic acid, whereas the second group includes sialylated glycans without a bisecting GlcNAc. In addition to aging, high levels of glycans with a core fucose and bisecting GlcNAc can be found in the serum of patients with type 2 diabetes, which is similar to indicators associated with the risk of cardiovascular disease. It has now been found that sialylation of N-glycans reduces CVD (cardiovascular disease) risk but also has a negative feedback, with elevated levels of very low-density lipoproteins and triglycerides in serum and the presence of atherosclerotic lesions in the carotid artery [59].

Thus, we suggest changes in the relationship of the glycosylation patterns of immunoglobulins with various autoimmune, alloimmune, infectious, oncological, and other diseases and their association with a general inflammatory status. At the same time, apparently, B-cells have the ability to adjust the glycosylation profile of antibodies to increase antigen specificity, as well as to modulate effector functions.

## 3. Non-Enzymatic Glycosylation and Advanced Glycation End Products

Apart from post-translational glycosylation, non-enzymatic glycosylation (glycation) of circulating proteins should be mentioned as a completely different type of protein and glycoprotein modification, which is driven by chemical interaction of glucose and free amino groups of lysine residues. Such a modification can sufficiently affect the chemical and functional properties of proteins, including immunoglobulins. An important aspect is that glycation or “glycoxidation” of proteins culminates in the generation of advanced glycation end products (AGEs) [60,61]. AGEs are the heterogeneous group of compounds formed after a series of non-enzymatic reactions of proteins, lipids, and nucleic acids with a role in several pathologies such as atherosclerosis, diabetic microvascular, and macrovascular diseases, and metabolic syndrome [62,63]. The receptor for AGE (RAGE) is associated with RA due to its ability to induce inflammation. Takahashi et al. tagged acidic oligopeptide to a non-membrane bound form of RAGE (endogenous secretory RAGE (esRAGE)), and therefore assessed a bone-targeting therapeutic agent and therapeutic effectiveness in a murine model of CIA. D6-esRAGE administered weekly (1 mg/kg) to RA model mice was able to reduce inflammatory arthritis, synovial hyperplasia, cartilage destruction, and bone destruction. D6-esRAGE also reduced plasma levels of proinflammatory cytokines (TNF)-α, IL-1, and IL-6 [64]. Thus, it was shown that D6-esRAGE greatly facilitated drug delivery to bones. AGEs through RAGE can also stimulate proinflammatory mechanisms, therefore, increasing inflammatory effects in chronic inflammatory disorders [65]. Recent studies have suggested that AGEs increase during RA [60,61,66]. The decreased sRAGE levels could indicate activation of RAGE signaling and enhanced inflammation. Up to now, decreased serum level of sRAGE has been observed in RA [67,68], systemic lupus erythematosus [69], antiphospholipid syndrome [70], primary Sjogren’s syndrome [71], and multiple sclerosis [72]. Knani et al. indicated that patients with severe RA had lower serum sRAGE concentrations than patients with moderate RA and control subjects [68]. On the contrary, in the study by Jafari-Nakhjavani et al., higher serum sRAGE levels were revealed in RA patients as compared with healthy controls, which correlated positively with the disease stage [73]. In another study, Pullerits et al. indicated that blood and synovial levels of sRAGE were not associated with disease duration or acute-phase reactant C-reactive protein (CRP) [67].

In any case, non-enzymatic glycation is worth mentioning in the context of this review because it can exacerbate the damaging effects of deglycosylation of glycoproteins. For example, it has been shown that non-enzymatic glycation atop of desialylation of apoB-100, the major apoprotein of low-density lipoprotein, added much to its atherogenicity [74,75,76]; possibly, similar synergetic damaging effects could also occur in immunoglobulins.

## 4. Glycosylation Disorders in Rheumatological Diseases

Rheumatoid arthritis and primary osteoarthritis were the first diseases for which a connection with a change in the composition of IgG glycol was uniquely established. Patients suffering from these pathologies showed a higher level of agalactosylated IgG glycans than in the control group [77]. Further studies confirmed that the level of agalactosylated IgG glycans correlated with the clinical parameters of RA, such as the severity of symptoms, the activity of the disease, and its progression, as well as also for predicting the response of the disease to therapy [78,79,80,81].

The reduction of IgG galactosylation in RA correlated with interleukin (IL)-6 and C-reactive protein (CRP) [82]. With regard to IL-6, low concentrations of this cytokine can increase IgG galactosylation via modulation of glycosyltransferase activity, whereas high concentrations of IL-6 decrease glycosyltransferase activity [83]. This modulatory effect of IL-6 could explain why IgG galactosylation rates were decreased in RA [32].

Non-galactosylated (agalactosylated, G0) IgG aAbs have been associated with proinflammatory activity in patients with RA [32,82,84]. It has been shown that IL6ST-ANKRD55, the locus associated with RA development risk, and IgG agalactosylation were linked, as has been demonstrated in the population-based genome-wide association study (GWAS) [85].

Studies of antigen-specific antibodies in RA have shown that their glycans differed in antibodies to the cyclic citrulline-containing peptide (anti-citrullinated protein antibody, ACPA) of the IgG1 subclass from total IgG1 both in serum and in synovial fluid. The presence of ACPA in patients with differences in disease clinical course indicated important involvement of these autoantibodies in the pathogenesis of RA [86]. Glycosylation of Fc in IgG and ACPA in RA have been previously described [87]. Recently, it was demonstrated that the IgG Fc-region in ACPA was characterized by particular IgG subclass distributions and specific patterns of IgG1 Fc glycosylation [88,89]. The ACPA-IgG1 Fc region-derived IgGs differ from total IgG1s in terms of structure. Furthermore, mostly IgG1 and IgG4 react with CCP, citrullinated vimentin and citrullinated fibrinogen [89]. These features affect the IgG affinity to Fc receptors and complement, altering their functionality and pathway activity as a whole [90]. An investigation of the IgG Fc glycans in RA patients, revealed that the Fc sialylation levels in IgG and ACPA correlated with bone architecture. Bone volume decreased as IgG Fc sialylation levels were lowered, leading to bone erosion [91]. It is worth mentioning that ACPA are less sialylated than IgG, in general, which could be the reason for their high pathogenicity. This feature could explain the pro-osteoclastogenic effect of ACPA [92]. Sialylated ACPAs were unable to induce osteoclastogenesis. Furthermore, mice treated with the sialic acid precursor N-acetylmannosamine appeared to be less prone to bone loss due to inflammation, indicating that sialylated IgG was potentially a protective agent in this scenario [91].

It has also been demonstrated that ACPA glycoprofile was different in patients with positive and negative rheumatoid factor [84]. More importantly, the ACPA IgG1 glycoprofile began to change prior to disease onset [88].

The reduced IgG galactosylation was associated with other autoimmune diseases, such as systemic lupus erythematosus, inflammatory bowel disease, and vasculitis-associated antineutrophil cytoplasmic antibodies (antineutrophil cytoplasmic antibody-associated vasculitis), and also with the progression of these diseases, as well as the activity and severity of symptoms [38,93,94,95,96]. Impaired IgG galactosylation and sialylation prior to disease development characterizes ANCA-associated vasculitis [39].

Agalactosylated IgGs have been shown to mobilize the complement system more actively [82]. In turn, therefore, highly sialylated IgG played an important role in immune homeostasis and resisting inflammation and autoimmunity [3,97]. Recently, evidence has been obtained that active sialylation could be due to estrogen, which induces the expression of beta-galactoside alpha-2, 6-sialyltransferase 1 (St6gal1), which could explain the difference in the incidence of RA between women and men and among women before and after menopause [98].

## 5. Systemic Inflammation and Premature Atherosclerosis

Systemic inflammation contributes significantly to the development of premature atherosclerosis in RA. It has been established that the main cause of high premature mortality in immuno-inflammatory RA was associated with cardiovascular complications (CVC) caused by accelerated progression of atherosclerosis and the development of chronic heart failure. Most often, CVC develope in patients with RA with low or moderate cardiovascular risk, but with high clinical and immunological activity of the disease. Attention is drawn to the similarity of mechanisms of immunopathogenesis of RA and chronic low-grade inflammation in atherosclerosis. According to modern views, chronic inflammation associated with the uncontrolled activation of both innate and acquired immunity play a fundamental role at all stages of RA and the atherosclerotic process. Protein–glycan interactions play a crucial role in the relationship between pathogens and the immune system and altered IgG glycosylation affects its inflammatory properties [99]. Certain glycosylation patterns mean increased CVD risk. In a study of 27,941 participants in the Women’s Health Study, it was shown that GlycA (glycoprotein acetylation), a biomarker of plasma protein glycan N-acetyl methyl groups was related to the incidence of CVD which remained significant when adjusting for traditional risk factors and for CRP levels [100]. In a study by Menni et al., molecular pathways were observed that bound IgG with the formation of arterial damage [59]. Glycosylation features associated independently with subclinical atherosclerosis. Sialylated N-glycans in a glycosylation pattern correlated negatively with CVD risk, triglyceride (TG), and very low-density lipoprotein (VLDL) levels in serum.

AGEs are recognized as nontraditional risk factors that play crucial roles in increased cardiovascular mortality and morbidity in RA [101]. It is expected that, even in newly diagnosed RA, AGE accumulation and early stages of atherosclerosis, i.e., endothelial activation and dysfunction, could be present and that these changes could be reversed by reducing disease activity. AGEs can ligate to the receptor of AGE (RAGE), which, as it is present on endothelial cells (ECs), results in EC activation and formation of soluble vascular cellular activation molecule 1 (sVCAM-1) [102].

## 6. Immunoglobulin Fab and Fc Fragment Glycosylation

As stated above, immunoglobulins are the most important serum proteins that demonstrate changes in their glycosylation pattern; these changes can, in turn, affect their immunoeffector functions [25]. IgG modulates immune cell activity by interacting with FcγR, leading to effective employment of effector activities [91]. Different inflammatory factors can influence B cells during activation and differentiation, modulating the glycosylation of secreted IgG [103].

In healthy people, 15 to 25% of IgG Fab fragment is glycosylated [104]. Nevertheless, the glycosylation is more, even in this case, with a more limited glycan profile than in the Fc domain. However, with regard to the role of sialylation of F(ab’)2, various effects have been noted depending on the model system understudy.

A change in the degree of glycosylation of IgG Fab fragment is also noted in human pathological conditions. For example, an increase in the carbohydrate content in the Fab fragment of autoantibodies with rheumatoid arthritis, up to 80%, was recorded with a simultaneous two-fold decrease in the ability of autoantibodies to bind antigen [105]. In B-cell lymphoma, the appearance of new sites of N-glycosylation of the Fab fragment was detected. N-glycosylation of Fab fragment modulates the ability of IgG to bind antigen, and affects the half-life of antibodies, the ability of antibodies to aggregate, and the formation of immune complexes [106]. The mechanism of the influence of glycans on these properties of antibodies is unclear. In most cases, to explain these effects, researchers have proposed a hypothesis about the effect of the carbohydrate residue on the conformation of the variable region of the antibody.

Models of inflammation in an in vitro experiment that stimulated lipopolysaccharide (LPS) or phytohemagglutinin (PHA) revealed that removing sialic acid residues from F(ab’)2 fragment reduced the anti-inflammatory activity of the antibodies [107]. Sialylation of F(ab’)2 played an important role in modulating cytokine secretion by plasmacytoid dendritic cells (pDCs). Thus, F(ab’)2 fragment enriched in sialic acid stimulated the monocytes-mediated E2 prostaglandin production that inhibited the release of IFN-α [108].

In comparison to the Fab fragment, much more research has been devoted to glycosylation of the IgG Fc fragment, due to the possibility of modifying the functional activity of therapeutic antibodies by changing the glycosylation profile of the Fc domain. The structure of the Fc fragment of human IgG allows the antibody to perform its effector functions, i.e., act as an inducer of phagocytosis, participate in the implementation of the antibody-dependent cell cytotoxicity (ADCC), complement-dependent cytotoxicity (CDC), antibody-dependent cell phagocytosis (ADCP) programs, and thus modulate anti-inflammatory activity [109]. Humans have six classic receptors interacting with the Fc domain (FcgRI, FcgRIIa, FcgRIIb, FcgRIIc, FcgRIIIa, and FcgRIIIb). They are found in various combinations in all cells of the innate immune system [110].

The IgG Fc sugar moieties, known as N-glycans, affect the affinity of the Fc domain in FcRs and complement factors, ultimately initiating to an array of inflammatory responses. Glycosylation of IgG Fc N-glycans is altered during pathological or changed physiological conditions, which influences structure and stability of aAbs [1,2,111].

A pregnancy-related phenomenon of IgG galactosylation has been observed in healthy women and women with chronic inflammatory disease such as rheumatoid arthritis (RA). In a detailed study of Fc glycosylation in IgG, during and after pregnancy, IgG galactosylation increased gradually during gestation, peaked in the third trimester, and decreased postpartum in healthy women and in RA patients. In these patients, increased Fc galactosylation in IgG correlated with overall improvement during pregnancy, whereas postpartum flare was associated with decreased galactosylation of Fc in IgG [32].

IgG glycans rich in sialic acid can modulate the survival and activation of B cells. This process is mediated by signaling through a CD22 receptor on B cells surface. Researchers of this mechanism have found that apoptosis of B cells was enhanced in the presence of intravenous immunoglobulin (IVIg). This proved the modulation of the adaptive immune system depending on the level of sialylation of IVIg [112].

The use of artificially sialylated Fc fragments has confirmed the assumption of anti-inflammatory activity of antibody sialylation [14]. The mechanism of this effect is not fully understood. There are several likely explanations. One model suggested that sialylation sterically limited the region of the Fc fragment, decreasing the affinity of the antibody for classical Fc receptors, while increasing the affinity for the nonclassical Fc receptor DC-SIGN (dendritic cell-specific intercellular adhesion molecule-3-grabbing non-integrin, CD209). It has been shown that DC-SIGN binding controlled the cascade triggering the synthesis of interleukin-33 dendritic cells, IL-4 basophils, which ultimately led to activation of FCgRIIb on macrophages, thereby nonspecifically inhibiting their proinflammatory activation [15,113]. Other likely patterns including receptors such as Siglec/CD22 [112], C-type lectin receptors [114], and Fc receptor-like protein 5 [115], by binding to these sialylated structures, are capable of stimulating an anti-inflammatory effect.

A significant addition is the data on the change in age of the content of agalactosylated forms of Fc IgG fragment in human serum. As levels of G0 forms increase, and the proportion of sialylated forms decreases [17]. Interestingly, pregnancy-induced galactosylation and sialylation of IgG improve the course of rheumatoid arthritis [32]. There is also a decrease in galactose in systemic vasculitis, such as granulomatosis with polyangiitis and microscopic polyangiitis. The terminal sialylation of IgG glycan can be considered to be a protective reaction in autoimmune diseases [116]. Thus, the introduction of sialylated glycan IgG into the body has a powerful anti-inflammatory effect that can be used as the basis for the creation of glyco-modified therapeutic monoclonal antibodies.

## 7. Various Uses of Modified Immunoglobulins in the Treatment of Certain Diseases

Glycosylation changes in IgG proteins isolated from the sera of patients with inflammatory arthropathies possess the potential to be crucial elements for the development of new therapeutic strategies [34]. Highly-galactosylated and highly-sialylated IgG antibodies can inhibit a proinflammatory immune response in an antigen-specific manner and could be a key for regaining tolerance against defined self-antigens in autoimmune patients [117].

Currently, immunoglobulins and Fc fragments are used in the treatment of certain diseases, for example, thrombocytopenic purpura (ITP) [118], rheumatoid arthritis [119], and nephrotoxic nephritis [120]. A study of Fc glycosylation in inflammatory arthritis, ITP, and epidermolysis of bullosa acquisita (EBA) models showed that when sialic acid was removed from Fc fragments, it made IVIg unable to exhibit anti-inflammatory activity [8]. Conversely, anti-inflammatory activity could be increased after sialylation of terminal glycan residues associated with the Fc fragment [8].

The anti-inflammatory effects of sialylated IgG aAbs were first reported for the intravenous immunoglobulin (IVIg). IVIg is a complex heterogeneous mixture of human antibodies (primarily polyclonal IgG) and non-IgG proteins that is indicated for use as an immune replacement therapy in patients with primary and secondary immune deficiencies, and as an immunomodulatory treatment for several autoimmune and inflammatory diseases. Human IgG, which is composed of IgG1, IgG2, IgG3, and IgG4 subclasses, is one of the most dominant and significant glycoproteins in human serum [121]. Since these regions were involved in the binding of IgG to Igs-Fc receptors and complement component 1q (C1q), each subclass of IgG showed differential functions with respect to antigen binding, immune complex formation, complement activation, triggering of effector cells, half-life, and placental transport [6].

Further studies have described that the anti-inflammatory activity of IVIg was associated with a certain group of IgG molecules with α-2,6-sialic acid residues on polysaccharides linked with Fc [43]. Such a polysaccharide structure was found only in 1–3% of IgG molecules in IVIg preparations, corresponding to anti-inflammatory activity of IVIg in high doses. Recent studies have shown that the anti-inflammatory properties of IVIg could be fully reproduced using a recombinant drug corresponding to sialylated IgG Fc fragment [43]. Such a recombinant drug had 35 times higher in vivo activity and, accordingly, it could be used at much lower doses than IVIg preparations. For the manifestation of the anti-inflammatory effect, sialylated IgG Fc fragment required the expression of a special C-type lectin, the so-called SIGN-R1 substance, on macrophages in the marginal zone of the spleen [122,123].

The sialylated IVIgs modulate arthritis in mice via binding to the SIGN-R1 receptor (specific ICAM-3 grabbing non-integrin-related 1) [90] and in this way upregulate the inhibitory Fcγ receptor FcγRIIB on effector macrophages [15]. Furthermore, sialylated IVIgs inhibit the maturation of dendritic cell via the FcγRIIB-independent pathway [114]. Thus, there is evidence that sialylated IVIgs are anti-inflammatory agents in the case of targeting innate and adaptive immune cells.

When applied, sialylated IgG autoAbs in small doses reduced joint swelling in a CIA mouse model [12]. Endogenous sialylation of IgG aAbs slowed down arthritis and nephritis progression in mouse models via an IVIg-like pathway [124]. Finally, immune complexes (ICs) with sialylated antigen-specific IgG aAbs inhibited IL-6 production by dendritic cell in vitro [125]. ICs containing autoantigen-specific sialylated IgG aAbs affected activation of DCs and the production of IL-6 independently of FcγRIIB and ameliorated autoimmune pathologic states [126].

Kaneko et al. found that the anti-inflammatory and proinflammatory activities of IgG and Fc came from differential sialylation of the carbohydrate chains of the Fc region [8].They reported that administration of IVIg to mice inhibited inflammatory responses induced by intravenous injection of K/BxN serum, which was considered to be a model of RA. Furthermore, sialic acid enriched IVIg, obtained by collecting the binding fraction of IVIg to a *Sambucus nigra* agglutinin (SNA)-agarose column, expressed much higher anti-inflammatory activity than the original IVIg. It was also found that injection of the Fc fragment, but not the Fab fragment of IVIg, induced the anti-inflammatory activity, and the SNA binding fraction of the Fc fragment was 10 times more active than the intact Fc fragment. Accordingly, IgG acquires anti-inflammatory properties upon Fc sialylation. Kaneko et al. also showed that the Fc sialylation of IgG was reduced by the induction of an immune response. Therefore, this mechanism can be characterized as a turning point from anti-inflammatory to proinflammatory activity.

Guo et al. showed that repeated injection of ovalbumin in mice caused production of specific IgGs and an increase of fucose content in the N-linked carbohydrate chains of the IgGs [127].

Some of the most effective treatments for inflammatory arthropathies involving anti-TNF therapy affect the IgG glycosylation status. The effects of infliximab on IgG glycosylation status, in patients with CIA, RA, and spondyloarthropathy (SpA) have been studied recently [128,129]. The effect of methotrexate in combination with anti-TNF therapy has also been studied in patients with RA [130,131].

There is a strong correlation between serum glycosylation in patients with RA and SpA and inflammation, as changes in patients’ serum glycome is a measure of the anti-TNF effect on the immune system [132]. Elevated galactosylation of IgG glycans means formation of a “healthy” phenotype, and an overall decrease of inflammation, since the shift in glycosylation correlates with lower levels of CRP.

## 8. Conclusions

The active use of drugs based on immunoglobulins and their derivatives poses new challenges for specialists at all levels. Understanding the mechanism of the underlying therapeutic effects is impossible without studying the basic principles of the functional effects of these molecules on a particular physiological aspect and the pathophysiology of disease. Thus, the post-translational modification (glycosylation) of immunoglobulins expands the possibilities in the diagnosis of both immunological and inflammatory disorders and in their therapies. Known data indicate that this process is actively controlled at the molecular level and plays one of the determining roles in the regulation and functional activity of antibodies. However, the mechanisms underlying this control are not fully understood. Future studies should focus on the study of signal transmission during the production of immunoglobulins by B cells, which would help to unravel the mechanisms underlying the specific glycosylation of antibodies. This would establish the keyways to realize the effector activity of antibodies. Thus, obtained data would give new impetus to the development of therapeutic drugs and vaccines for the next generation, i.e., the next chapter in the treatment of a wide range of infectious, inflammatory, oncological, and autoimmune diseases.

## Abbreviations

aAbs	Autoantibodies
ACPA	Anti-citrullinated protein antibody
ADCC	Antibody-dependent cell cytotoxicity
ADCP	Antibody-dependent cell phagocytosis
AGEs	Advanced glycation end products
CDC	Complement-dependent cytotoxicity
CVD	Cardiovascular disease
CEL	N^ε^-(carboxyethyl) lysine
CDRs	Complementarity-determining regions
CIA	Collagen-induced arthritis
CRP	C-reactive protein
CVC	Cardiovascular complications
CVD	Cardiovascular disease
DC	Dendritic cells
EBA	Epidermolysis of bullosa acquisita
ECs	Endothelial cells
Ig	Immunoglobulin
IVIg	Intravenous immunoglobulin
IL	Interleukin
ICs	Immune complexes
ITP	Thrombocytopenic purpura
HIV	Human immunodeficiency virus
LPS	Lipopolysaccharide
MBL	Mannose-binding lectin
MG	Methylglyoxal
PHA	Phytohemagglutinin
pDCs	Plasmacytoid dendritic cells
RA	Rheumatoid arthritis
sVCAM-1	Soluble vascular cellular activation molecule 1
SpA	Spondyloarthropathy
TG	Triglyceride
TLA	Three letter acronymn
TNF	Tumor necrosis factor
VLDL	Very low-density lipoprotein
